# Scientific Lectures for the Insane

**Published:** 1845-03

**Authors:** 


*Scientific Lectures for the Insane.*—Dr. Earle, in his recent report of the Bloomingdale Asylum, which we have read with much satisfaction, has really made known a new source of rational enjoyment for lunatics. He has been giving a series of scientific lectures, generally illustrated by diagrams and pictures, of a size to enable every person in attendance to have a distinct view of them. Among other subjects, Dr. Earle lectured on the physiology of the eye, and the phenomena of vision; physiology of the muscular system; and the following are to be the ensuing topics: physiology of the brain and nerves; heart and blood vessels, organs of respiration; auditory apparatus; organs of speech; electricity; hydrogen and nitrogen gases, &c. The result, on the disturbed minds of the Bloomingdale hearers, was most happy. "The several sources of instruction herein mentioned," says Dr. E., "are among the principal promoters of peace, tranquillity and order; they are some of the most valuable aids in restoring the mind to its original healthy action." Those who are placed over other lunatic institutions, have a very encouraging precedent to follow, and we fully expect to hear, in proper time, that an annual course of miscellaneous public lectures, expressly for the patients, is considered an indispensable curative process, conducing alike to present intellectual gratification and permanent enjoyment.—*Ib.*

